# Data Management Workflow for FAIR Sharing of Bioimaging Datasets in Plasma Medicine

**DOI:** 10.1038/s41597-026-07982-x

**Published:** 2026-08-04

**Authors:** Mohsen Ahmadi, Robert Wagner, Sander Bekeschus, Markus M. Becker

**Affiliations:** 1 Leibniz Institute for Plasma Science and Technology (INP), Felix Hausdorff-Str. 2, 17489 Greifswald, Germany; 2 Department of Dermatology, Venereology, and Allergology, Rostock University Medical Center, Strempelstr. 13, 18057 Rostock, Germany

## Abstract

Bioimaging experiments in plasma medicine generate datasets that extend beyond conventional imaging studies by combining microscopy data with heterogeneous metadata from biological experiments and gas plasma treatments. These data are often distributed across multiple systems, making it difficult to maintain links between imaging data, plasma treatment conditions, biological metadata, and microscopy acquisition settings. This fragmentation limits data sharing and repository deposits. To address this challenge, we present a data management workflow for FAIR sharing of bioimaging datasets in plasma medicine. The workflow is implemented as an open-source Jupyter Notebook and connects the established research data management tools Open Microscopy Environment Remote Objects (OMERO) for image data management, eLabFTW as an electronic laboratory notebook for experimental documentation, Adamant as a schema-based metadata acquisition tool, and Micro-Meta App for the standardized capture of microscopy settings. The workflow guides researchers through the data management process, supporting the adoption of the FAIR data principles for the sharing and reuse of imaging datasets. We demonstrate how biological metadata, plasma-treatment parameters, microscopy settings, and image data are associated across the Screen, Plate, and Well hierarchy in OMERO through structured JSON metadata records generated in Adamant, stored in eLabFTW, and linked to imaging datasets. By means of a structured FAIR assessment, the contribution of the workflow components to the achievement of FAIR imaging datasets in plasma medicine is demonstrated, resulting in a FAIR compliance level of 50–60%. Thus, the Jupyter Notebook workflow supports researchers in plasma medicine and other domains with similar requirements by linking image data and metadata to experiment descriptions, enabling FAIR sharing and simplified reuse of bioimaging datasets.

## Introduction

Plasma medicine is an interdisciplinary field that explores the application of cold gas plasma (a partially ionized gas containing free electrons, ions, and reactive species^1^) for therapeutic and biomedical purposes^2,3^. It combines principles from plasma physics, biology, medicine, and engineering to develop novel treatments and diagnostic tools for various diseases. Microscopy-based imaging is one of the main pillars of experimental research in plasma medicine, offering insights into how cells and molecules respond to plasma treatment, which guides the study of gas plasma-induced biological effects^4–6^. These processes are dynamic and often require quantitative time-resolved imaging to capture changes across varying conditions, plasma exposure times, and biological models, thereby supporting a mechanistic understanding of plasma-target interactions. As image acquisition technologies advance toward high-throughput and high-content formats, establishing reproducible bioimage workflows has become essential for integrating plasma medicine research into the global bioimaging community, which is actively developing standardized metadata practices^7,8^. However, research data management (RDM) in microscopy is challenged by the large volume and heterogeneity of image data and metadata. Experimental records, raw imaging files, and metadata are often stored in separate systems, such as laboratory notebooks for protocols and metadata and image repositories, such as Open Microscopy Environment Remote Objects (OMERO), for raw data^9^. This fragmentation hinders reproducibility, transparency, and traceability, ultimately limiting compliance with the F(findable)A(accessible)I(interoperable)R(reusable)-data principles, which provide a guiding framework for improving the management and stewardship of scientific data^10^. By establishing semantic linkages between raw data, metadata, and analytical outputs across experimental stages, a data management workflow for bioimaging lays the groundwork for interoperable RDM infrastructures that support data sharing and reuse in imaging-related plasma medicine studies.

Microscopy plays a central role in plasma medicine by enabling the detailed visualization of how cells respond to plasma-induced effectors, such as reactive species. Reactive species can influence key biological processes such as apoptosis, cell proliferation, and oxidative signaling. To investigate these effects, researchers commonly employ fluorescence microscopy^11^, live-cell imaging in multi-well formats^12^ (e.g., time-lapse imaging of multiple conditions within a single plate), drug screening and dose-response assays across defined compound panels^13^, and automation-assisted fluorescence imaging^14^ of multi-well plates, where individual wells correspond to specific plasma treatments, genetic perturbations, or experimental conditions. Such automation provides systematic image acquisition across many wells while maintaining controlled experimental variability, but only becomes high-content imaging or screening when combined with multiparametric feature extraction and large-scale automation^15^. These complications create distinct and multifaceted challenges, as multiple large sets of structured and unstructured data, including images, protocols, reagents, treatment parameters, and analysis outputs, must be consistently linked, contextualized, and made accessible. Beyond local data management, an important objective of bioimaging workflows is to support the generation of datasets that can be shared, reused, and integrated across different studies. These datasets may be shared via community repositories, such as the BioImage Archive (BIA) and Image Data Resource (IDR), which support the long-term preservation, accessibility, and reuse of bioimaging data^16,17^. In addition, plasma science/plasma medicine datasets can be deposited in the INPTDAT (https://www.inptdat.de/) research data repository to support sharing and reuse^18^. Imaging data associated with a publication can be deposited in community repositories; however, such deposition typically requires standardized file formats, structured metadata, and clear provenance information, which are often not yet established in plasma medicine research.

**Fig. 1:**
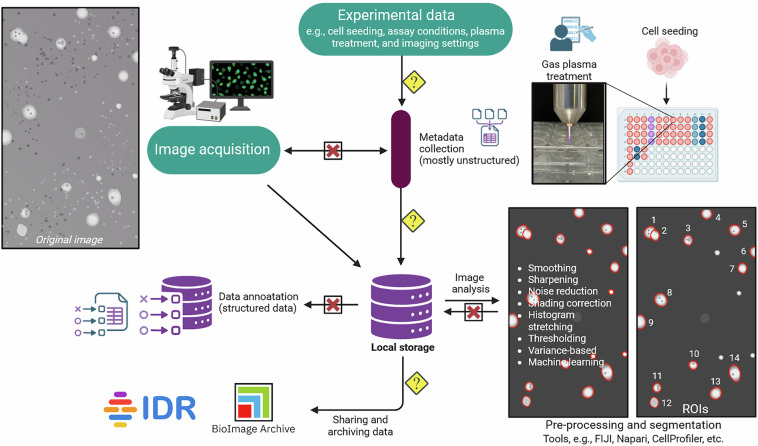
Overview of a traditional example of local bioimaging data management Image acquisition produces priandry raw image data along with the experimental parameters (cell seeding and gas plasma treatments) captured both during setup and during imaging, yet the associated metadata collection remains largely unstructured. Data are typically stored locally, annotated manually, and processed using analysis tools. The resulting datasets, often fragmented across files and folders, may create problems with sharing and archiving open repositories such as IDR and BIA. (X) Missing or manual data transfers; (?) Indicate uncertainties, such as incomplete metadata and unclear storage procedures. Created in BioRender. Bekeschus, S. (2026) https://BioRender.com/6tae47g.

Despite its importance, plasma medicine lacks FAIR-aligned RDM practices tailored for imaging. Imaging workflows in plasma medicine still face traditional RDM challenges, including unstructured metadata, manual and error-prone data handling, poor linkage between images and experimental parameters, and the use of proprietary or non-standardized formats (Fig. 1).

As shown, image acquisition and experimental metadata collection occur separately, resulting in heterogeneous and often incomplete metadata sets. Image analysis generates derived data that are not systematically linked to raw images. Local storage functions as a central repository but lacks standardization, version control, and traceability. Several tools have been developed to address the broader challenges of microscopy data handling. OMERO is one of the most widely adopted solutions, providing centralized data storage, access control, metadata annotation, and integration with analysis workflows, capabilities that benefit facility staff and end users^19^. OMERO is widely adopted by imaging facilities^19^ to standardize metadata handling, data organization, and reproducible workflows, demonstrating its applicability for institutional-scale bioimaging data management and sharing^20^. IDR are built on the OMERO infrastructure and application programming interfaces (APIs), providing mechanisms for data submission, visualization, querying, and access to curated image datasets^17^. The BIA supports broad life-science microscopy data deposition and uses the Open Microscopy Environment (OME) ecosystem, including OME metadata standards and formats, as a key component of its data ingestion and metadata workflows^16^. The open-source image data management platform OMERO supports local image management and provides a technological foundation for dataset publication and sharing. By preserving image provenance, annotations, and metadata relationships, OMERO facilitates the preparation of reusable datasets that can be transferred to institutional^19^ or community repositories^17^. Building on OMERO’s capabilities, Massei *et al*.^21^ have developed semi-automated workflows for the combination of OMERO with workflow management systems to align high-content screening bioimaging data management with FAIR principles. Complementary to this, Escobar Díaz Guerrero *et al*.^22^ have introduced LEO, a platform designed to link OMERO with electronic laboratory notebooks (ELNs) and other metadata. LEO offers retrospective integration of the experimental context with imaging datasets by linking image data to metadata from ELNs and metadata management systems, thereby supporting richer contextualization.

In addition to these integration platforms, a range of specialized tools supports the standardization of individual (meta)data commonly encountered in bioimaging workflows. The Micro-Meta App supports the consistent documentation of microscope hardware configurations and image acquisition parameters in a machine-readable format^23^. It complements metadata automatically extracted from image files using Bio-Formats by capturing hardware configurations, acquisition settings, calibration information, and other contextual details that may not be consistently available in vendor-specific image formats. ELNs such as the open-source systems eLabFTW^24^ (https://www.elabftw.net/) and openBIS^25^ (https://openbis.ch/), as well as the commercial product LabArchives (https://www.labarchives.com/), support the standardized documentation of experimental procedures. The open-source software Adamant extends this coverage to the entire bioimaging workflow, from sample preparation and treatment conditions to data capture, generating structured and machine-readable records that complement imaging metadata^26^. Jupyter Notebook (https://jupyter.org/) is widely used for transparent and reproducible workflows as a central interface by combining executable code, documentation, workflow components, and APIs within a single interactive environment^27^. For example, several Jupyter-based workflow implementations have been reported for metadata exchange and the integration of heterogeneous research data sources^28–30^.

To achieve an applicable RDM framework for plasma medicine, key aspects, including metadata collection and standardization (Adamant, Micro-Meta App, and eLabFTW), metadata exchange (using APIs and machine-readable records), dataset annotation and image management (OMERO), and FAIR data management across the entire data lifecycle, must be integrated into a unified workflow that reflects the complexities of biological imaging experiments and plasma medicine research. Several initiatives have emerged to address this. The NFDI4BIOIMAGE consortium (https://nfdi4bioimage.de/) is developing a national infrastructure to support standardized and interoperable bioimage data across institutions^31^. The Recommended Metadata for Biological Images (REMBI) offers tiered guidelines for reporting metadata in biological imaging^32^, and minimum information guidelines now support standardized documentation for modalities such as 3D microscopy, multiplexed tissue imaging, and cell migration experiments^15,33^. Plasma-MDS^34^ is a plasma-specific metadata schema integrated into NFDI4BIOIMAGE to enable domain-relevant metadata annotation, including imaging-based studies^31^. Despite these advances, adoption in plasma medicine remains limited, with imaging workflows often relying on inconsistent documentation and ad hoc data handling, which hinders alignment with the FAIR principles. To address this gap, we introduce a data management workflow for bioimaging studies in plasma medicine that integrates OMERO, eLabFTW, Adamant, and Micro-Meta App in order to link experimental/technical context (including imaging, biological, and gas plasma metadata), instrument settings, and imaging data. The aim is to achieve FAIR imaging datasets that are ready for sharing and simplified reuse. The workflow is implemented in an interactive Jupyter Notebook that integrated executable code, documentation, visual outputs, and user interaction.

## Methods

The workflow is developed using a user-centered and FAIR-by-design approach to address the requirements of bioimaging research in plasma medicine. Our target user group and their experimental practices are analyzed to identify the requirements for (meta)data management. Based on these requirements, a workflow integrating metadata collection, electronic laboratory documentation, image data management, and automated metadata linkage is designed and then implemented using open-source software components. Finally, the workflow is evaluated with respect to its compliance with the FAIR principles and its adoption in plasma medicine research.

### Defining the target group and workflow approach

To develop a user-friendly workflow, we first examined the primary producers of bioimaging data in plasma medicine and analyzed how image data and associated metadata are generated in the entire experimental process. Our target users are researchers who culture cells or prepare tissues, apply gas plasma treatment as an experimental intervention, and acquire microscopy images before and/or after treatment. Although these users possess deep domain knowledge, they often lack experience in RDM. The workflow is therefore designed to address their specific needs by providing data (images) and metadata management, automated linkage of experimental metadata to image data, and support for FAIRification and publication-ready datasets. To keep the information manageable, metadata are organized across different levels, allowing users to access only the details relevant to their roles, as defined by the team leads and project scope.

### Metadata collection and schema management

Metadata collection in the workflow is based on standardized, schema-driven approaches designed to operationalize FAIR principles. Metadata is exported in JavaScript Object Notation (JSON), a lightweight machine-readable format for data exchange between software systems while preserving metadata consistency and interoperability. Handling of experiment metadata is supported by Plasma-MDS (https://github.com/plasma-mds/plasma-metadata-schema)^34^. Plasma-MDS is a metadata schema that structures the description of plasma experiments based on the JSON schema, including all relevant device and process parameters and diagnostics. Metadata is captured and validated as JSON files that are stored and linked to experimental records in an ELN. Schema definitions and metadata templates are version-controlled and support the integration of schema-driven metadata acquisition (e.g., REMBI-compatible Excel templates for biological metadata collection^35^) into routine laboratory practices.

### Technical infrastructure and software components of the proposed workflow

The workflow is implemented using open-source software components. OMERO, developed by OME, provides a client-server platform for managing, visualizing, and annotating biological imaging data^9,36^. OMERO.server serves as the central data management component, whereas user interaction is provided by OMERO.insight, a desktop graphical user interface for image import, and OMERO.web, a web-based interface for data access, visualization, and annotation^9,36^. OMERO offers command-line tools and a Python API (omero-py), which support automation, batch processing, large-scale data import (e.g., high-content screening datasets), and implementation in external applications, including the Jupyter Notebook workflow described herein.

Metadata collection and validation are performed using Adamant^26^ (https://github.com/plasma-mds/adamant), a schema-driven metadata management platform. Adamant supports the generation and validation of JSON-based metadata records according to defined community standards and consistent metadata annotations using customizable templates. Adamant validates the collected metadata according to the corresponding JSON schema, checking the required fields, data types, and schema compliance before the metadata are exported as a JSON file. The resulting JSON record improves interoperability by providing machine-readable descriptions of the experiments. eLabFTW^24^ (https://github.com/elabftw/elabftw) offers human-readable experimental documentation and serves as a central platform for experimental records and associated metadata files linked to image data. The Micro-Meta App^23^ (https://wu-bimac.github.io/MicroMetaApp.github.io/) is used to document the microscope hardware configurations and acquisition parameters in a standardized and interoperable manner.

### Jupyter Notebook-based workflow implementation

In response to the requirements of the target group, the workflow is designed as a practical data management solution for bioimaging in plasma medicine, integrating all workflow components within a single, user-friendly environment suitable for users with limited programming experience. Rather than introducing another standalone tool, the objective is to provide a unified interface that builds on established software components and combines documentation, metadata collection, and workflow execution, while allowing users to interact with existing platforms by means of their native APIs.

To achieve this, the workflow is implemented in a Jupyter Notebook providing executable code and step-by-step user guidance. The notebook is developed using Python 3.12.5 and can be executed, e.g., in a JupyterLab environment (https://jupyterlab.readthedocs.io/en/latest/). It automates key workflow steps, including biological metadata collection using REMBI-compatible Excel templates, transformation of metadata into JSON records, documentation of experiments in eLabFTW, annotation of microscopy datasets in OMERO, and linking image data with experimental metadata using persistent identifiers (IDs). Because access to the OMERO server, eLabFTW, and associated datasets requires user-specific authentication and permissions, the workflow is intended for execution within the users’ own computational environments. To run the workflow, users configure the host addresses of their OMERO and eLabFTW instances in the provided Python scripts, which manage authenticated communication, data transfer, and metadata synchronization between the two systems.

The complete workflow, including the Jupyter Notebook, source code, configuration files, metadata templates, API examples, processing scripts, and all required software dependencies, is hosted on GitHub (see Code Availability section) and published in the WorkflowHub^37^. The notebook implementation is version-controlled on GitHub, which allows users to obtain stable workflow releases, reproduce previous versions, and contribute to further developments.

### FAIR Assessment

The FAIR assessment is conducted in two steps. First, the workflow is evaluated according to the FAIR principles. Second, the Common DSW Knowledge Model^38^ (version 2.6.12; https://registry.ds-wizard.org/knowledge-models/dsw:root:2.6.12) is applied, which maps technical and organizational data management practices to the individual FAIR principles and their corresponding criteria.

## Results and Discussions

### Workflow Implementation

The implemented data management workflow operates across three interconnected levels^39^, namely metadata, data handling, and data processing.

I. **Metadata**. The experimental context, microscope setup, and image acquisition information are systematically documented using eLabFTW, Micro-Meta App, and Adamant. This system enables the standardized capture of metadata and its linkage to image datasets using workflow components.
II. **Data handling**. OMERO serves as a central platform for image management. It secures batch uploads, integrates metadata from eLabFTW, and ensures long-term data accessibility.
III. **Data processing**. The workflow enables the batch analysis of fluorescence images from multi-well plates and supports customizable image analysis pipelines, allowing flexible adaptation to specific experimental requirements.

**Fig. 2:**
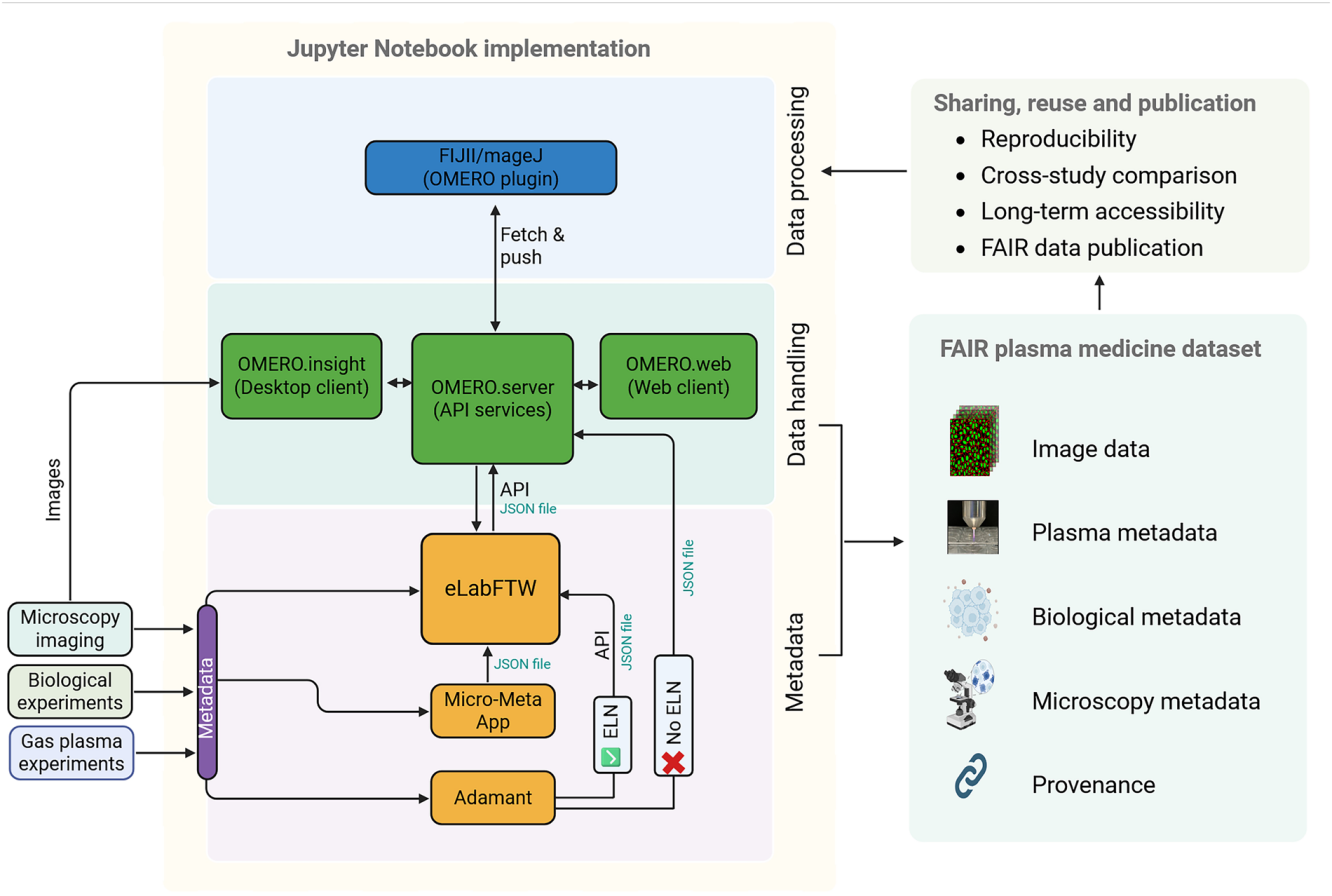
Overview of the data management workflow for bioimaging in the plasma medicine. The workflow is organized into three interconnected levels: metadata management (Micro-Meta App, eLabFTW, and Adamant), data handling (OMERO), and data processing (image analysis). Image data, microscopy metadata, biological metadata, and plasma-treatment metadata are collected and integrated by the respective software systems. All workflow components are connected via APIs within a Jupyter notebook-based workflow implementation guiding researchers through the RDM process. The resulting output is a dataset that combines imaging data with experimental and technical metadata, ready for sharing and reuse. Created in BioRender. Bekeschus, S. (2026) https://BioRender.com/1s1dfpy.

Figure 2 illustrates these three levels, their technical (software) components, and the information flow between them. It also highlights the workflow outputs, including the sharing, reuse, and publication of plasma medicine datasets. The workflow levels are described below, and the resulting dataset, FAIR assessment, and data-sharing aspects are discussed in subsequent sections.

### Metadata

**Fig. 3:**
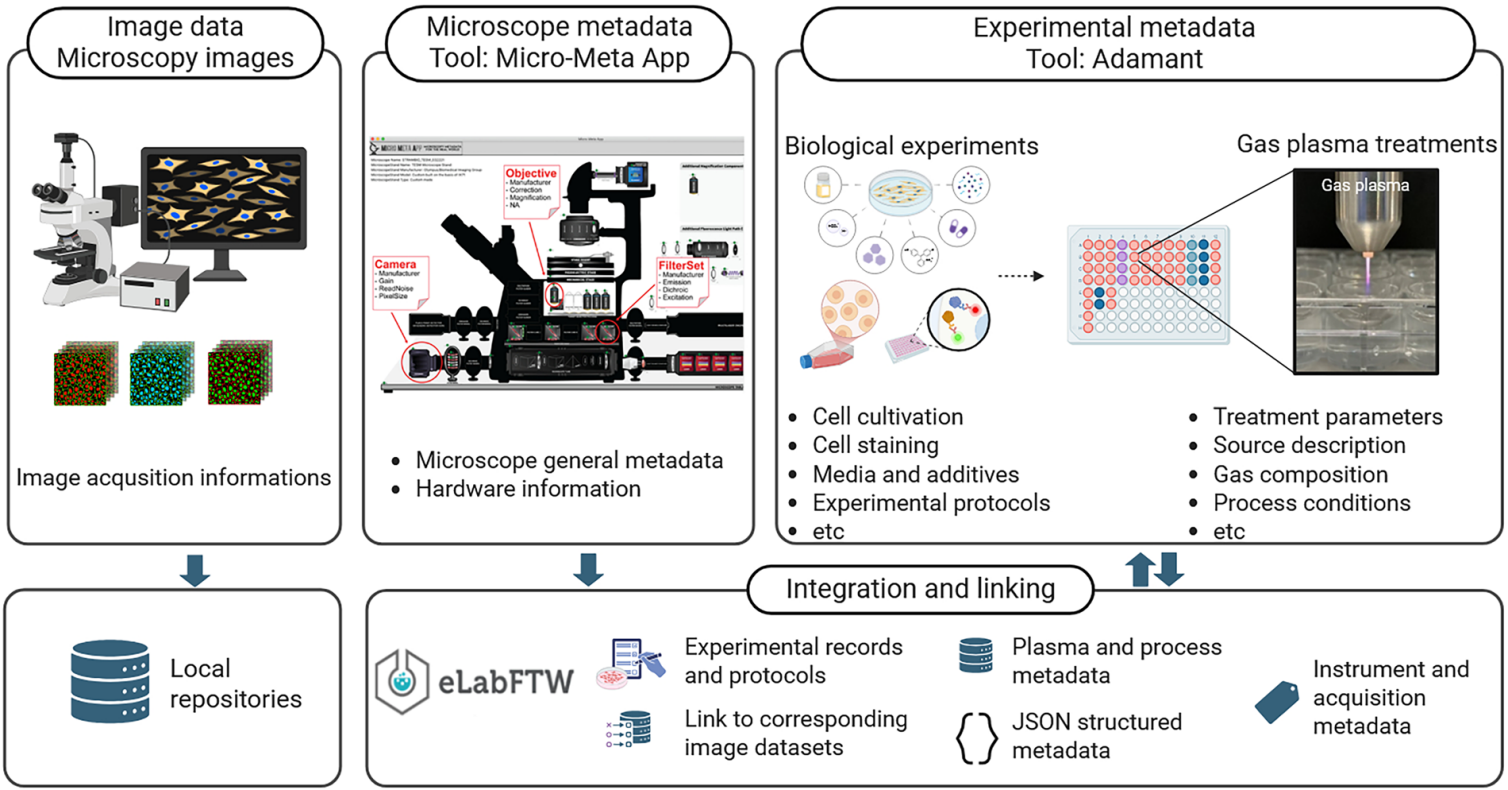
Metadata collection and integration within the plasma medicine bioimaging workflow. Metadata are collected from three complementary sources: microscopy images and acquisition information, microscope hardware configurations and imaging settings documented using Micro-Meta App, and experimental records describing biological assays and gas plasma treatments captured using Adamant. The resulting metadata are integrated and linked within eLabFTW. Created in BioRender. Bekeschus, S. (2026) https://BioRender.com/qlvnze6. Micro-Meta App image is adopted under the terms of the Creative Commons CC-BY 4.0 from Ref. ^23^.

As the first stage of the workflow, a metadata level is established to capture all experimental aspects in a reusable and machine-actionable manner. Metadata is systematically recorded across the entire experimental life cycle, including experimental planning and objective definition, protocol specification, sample preparation, plasma treatment, downstream biological assays, and microscopy imaging. Figure 3 shows an overview of the metadata level of the workflow, indicating how information from microscopy images, microscope metadata, and experimental metadata, including biological experiments and gas plasma treatments, is collected and integrated. The metadata are organized into three complementary categories.

Biological experiments, which include detailed logging of reagents and additives (e.g., dyes and chemical treatments) with information on the source, lot number, and concentration. Biological materials (e.g., cell lines or primary cells) are annotated with provenance, passage numbers, and culture conditions. Gas plasma treatments cover plasma-specific parameters, such as exposure duration, gas composition, and environmental conditions during treatment. Microscope metadata document microscope hardware specifications and image acquisition parameters, including objective lens, exposure time, detector type and sensitivity (e.g., gain and binning), laser wavelength and power (for confocal/fluorescence), and Z-stack spacing or time intervals (for 3D/time-lapse imaging).

All metadata categories are documented and maintained within eLabFTW, where experimental records can be created, edited, and linked to the corresponding imaging datasets. Biological, plasma treatment, and microscopy metadata can be consolidated into structured JSON records, providing a unified description of the experimental context necessary for the interpretation, reproducibility, and reuse of published datasets.

Within the workflow, experimental data are organized according to the OMERO hierarchy of “Screen”, “Plate”, and “Well”. A “Screen” represents the overall experiment or study, typically comprising multiple plates that together define the complete experimental campaign (e.g., plasma treatment or screening study). A “Plate” corresponds to a single physical multi-well plate (e.g., a 96- or 384-well plate) within a screen, defining the layout and grouping of experimental conditions. A “Well” represents an individual experimental unit within a plate, where a specific biological sample, treatment condition, and imaging configuration are applied and recorded.

The recorded metadata are collected using Adamant and stored in the corresponding eLabFTW experiment records. Adamant generates metadata according to the defined schemas, whereas microscope hardware specifications are documented separately using the Micro-Meta App. Biological experiment metadata are systematically collected using a REMBI-compatible Excel template^35^ and subsequently provided to Adamant as an attached file. The template is organized according to the hierarchy and records “Well”-level metadata within the context of the corresponding “Plate” and “Screen”. The template captures the sample identifiers, imaging channels, biological targets, experimental variables, and gas plasma treatment parameters. This template produces machine-readable metadata by recording “Well”-level biological, imaging, and treatment information for multi-well bioimaging experiments. It provides fields for identification (including well position and descriptive title), cell state at the time of imaging, and detailed per-channel imaging information. For each imaging channel, the template records the channel label, signal or contrast mechanism, and the targeted biological entity. Plasma-specific metadata, such as the plasma source, gas composition, treatment time, treatment distance, and treatment conditions, are captured in accordance with Plasma-MDS. In addition, optional extrinsic treatment variables can be documented, including treatment name, duration, solvent, concentration, and applied volume, depending on the requirements of the experiment.

In the next step, “Screen”- and “Plate”-level experimental metadata are collected via Adamant using a dedicated metadata schema (Fig. 3). The metadata schemas are aligned with established community standards, including REMBI^32^, ISA (Investigation, Study, Assay)^40^, MIHCSME (Minimum Information for High Content Screening Microscopy Experiments)^15^, and Plasma-MDS^34^, thereby facilitating their adoption for RDM in plasma medicine. This includes unique identifiers for the “Screen” and “Plate” constructed from institutional naming conventions and links to the corresponding eLabFTW experiment identifiers, as well as titles that match the respective objects on the OMERO server. Descriptive metadata, such as the author, description, protocol reference, and experimental dates, are recorded to provide contextual information. At the “Plate” level, biological and experimental descriptors are captured, including organism and cell type, accession numbers, and reference databases (e.g., ATCC or DSMZ), phenotype and genotype, culture conditions (media type, volume, concentration, and pH), seeding density, and plate-specific features, such as plate format, material, and well configuration. Additional metadata include the experiment type, imaging conditions (e.g., use of a lid, microscope name), plate preparation date, and creator information. Finally, the recorded REMBI-compatible Excel template is uploaded and linked within Adamant, enabling the integration of “Screen”, “Plate”, and “Well” metadata into a unified representation. The resulting JSON file created using Adamant is then transferred to the eLabFTW by means of API^24^, where it is stored as an attachment to the corresponding experiment record (Fig. 2). Before storing the JSON files in eLabFTW, Adamant validates the aggregated metadata based on the defined JSON schema.

Technical metadata related to imaging instrumentation is collected using the Micro-Meta App. Using the Micro-Meta App, key microscope and hardware specifications, including objectives, detectors, filters, light sources, acquisition modes, and calibration parameters, are systematically documented (Fig. 3). These details are essential for the accurate interpretation and reproducibility of microscopy data. The Micro-Meta App supports tiered metadata reporting levels as defined by the 4DN-BINA-OME initiative^41^ and exports metadata in JSON format, which is stored as an attachment to the corresponding experiment record in eLabFTW. Because microscope hardware specifications typically apply to the entire study, their metadata are annotated at the “Screen”, thereby avoiding redundancy across plates and wells.

It is important to mention that metadata transferred to OMERO through the workflow are immutable once created and can not be modified by the user. If metadata are revised in eLabFTW or by using Adamant and subsequently re-synchronized to OMERO, a new metadata record is generated rather than overwriting the existing annotation; both records are retained with their respective timestamps. This approach preserves provenance by maintaining a complete audit trail of metadata changes across systems and ensuring traceability of the original and updated experimental descriptions.

### Data handling

**Fig. 4:**
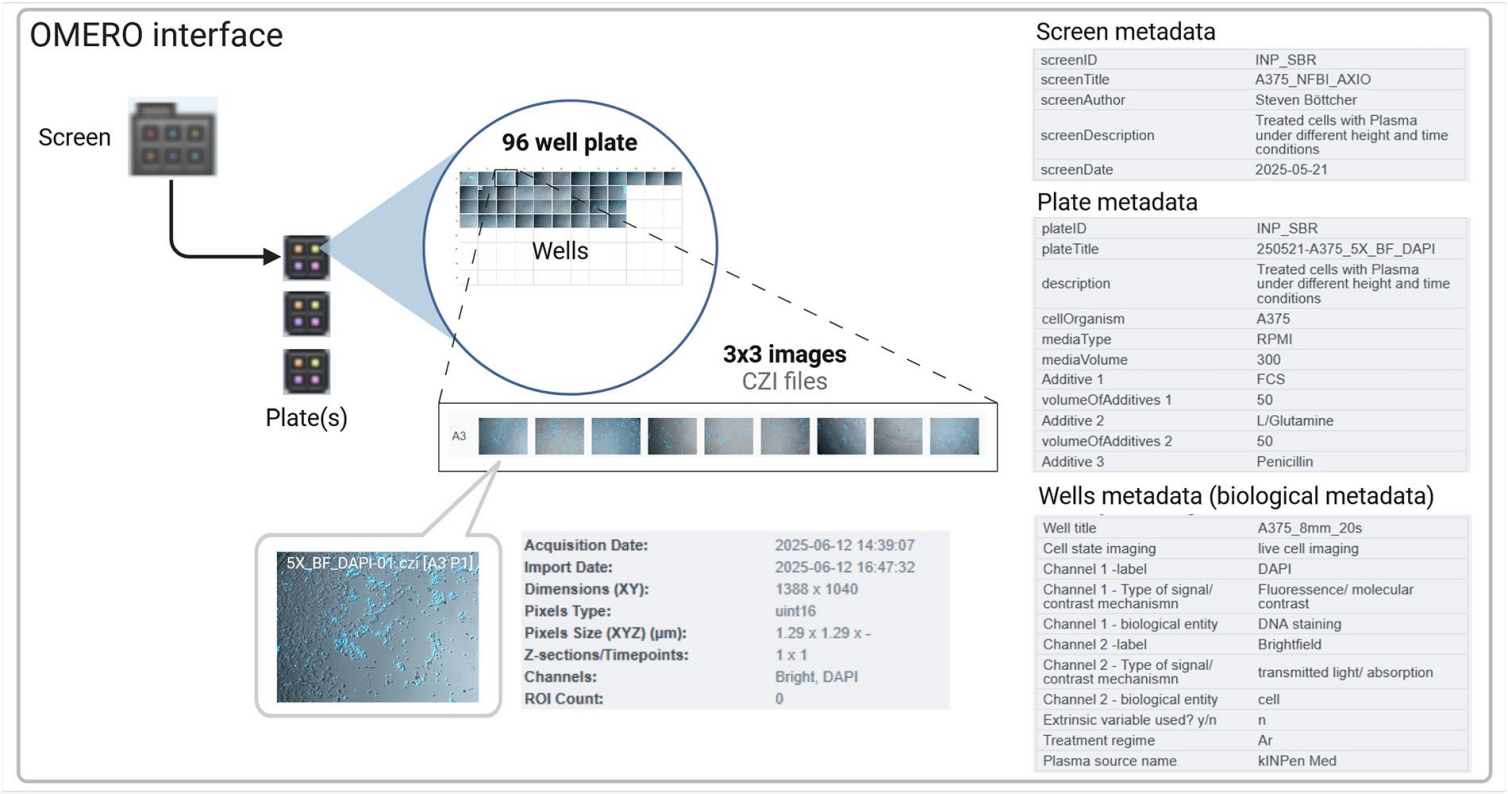
OMERO data organization and metadata annotation. (Left) Schematic representation of the OMERO data hierarchy used in automation-assisted fluorescence imaging. Experiments are organized into “Screens” comprising one or more multi-well Plates. Each “Plate” consists of individual “Wells”, and each well may contain multiple image fields (illustrated as a 3 × 3 tiled acquisition), reflecting automated microscopy workflows. (Right) Exemplary metadata annotations associated with the OMERO hierarchy, shown as key-value pairs (KVPs). Metadata can be assigned at different hierarchical levels (Screen → Plate → Well → Image) and are directly linked to the corresponding image data within the workflow. Created in BioRender. Bekeschus, S. (2026) https://BioRender.com/whuauio.

At the data-handling level within the workflow, OMERO serves as the central platform for microscopy imaging data and preserves image provenance, metadata, and annotations, which support the publication and reuse of datasets (Fig. 2). Raw image files from proprietary and open formats are ingested via Bio-Formats along with their associated acquisition metadata. Images are uploaded via OMERO.insight and organized according to the experimental design using “Screens.” Screens are used for automation-assisted fluorescence imaging workflows and represent multi-well formats typical of automated microscopy. Within the workflow, “Screens” organize images into Plates and Wells, reflecting the physical laboratory setup for cell cultivation. This hierarchical structure (Screens → Plates → Wells → Images) provides a flexible, experiment-aligned data organization within OMERO, as shown in Fig. 4.

When images are uploaded via OMERO.insight, the associated acquisition metadata are automatically extracted and transferred to OMERO together with the images, named as “Original Metadata”. Within the workflow, experimental metadata at the “Screen”, “Plate”, and “Well” levels, previously created using Adamant and the Micro-Meta App and stored as JSON files in eLabFTW, are programmatically imported into OMERO. This integration is implemented using Python functions based on the omero-py and elabapi-python libraries, which are executed in the Jupyter Notebook environment, connecting the OMERO server and eLabFTW by means of their respective APIs. Users enter the relevant Screen, Plate, and Well identifiers, together with the corresponding eLabFTW experiment IDs, into predefined fields provided by the Jupyter Notebook. Access to eLabFTW records is controlled via user-specific API tokens.

**Fig. 5:**
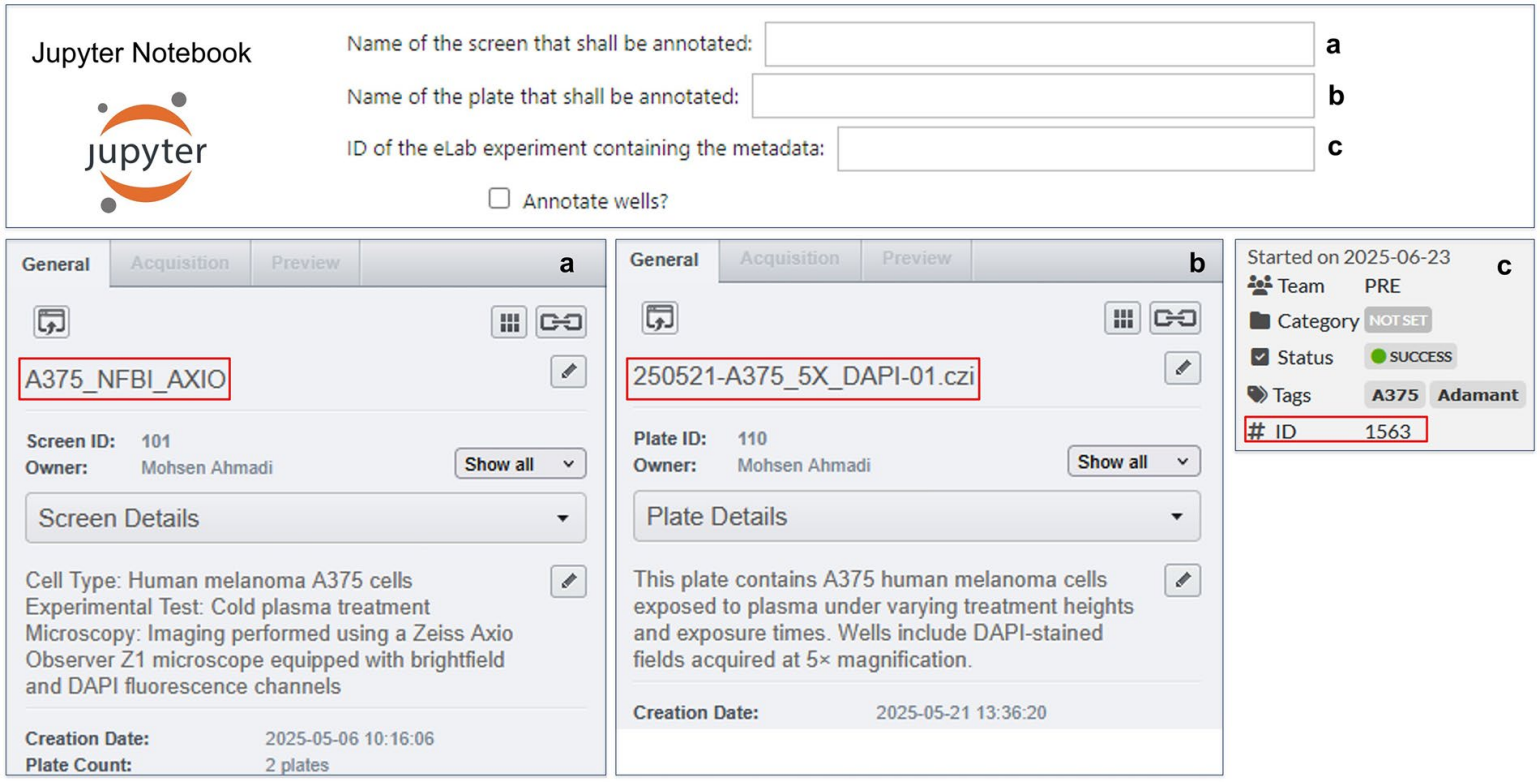
Jupyter Notebook interface for image data annotation on OMERO at the “Screens”, “Plates”, and “Wells”. (**a**) input field for specifying the “Screen” name to be annotated in OMERO, (**b**) input field for specifying the “Plate” name within the selected screen, (**c**) field for entering the eLabFTW experiment ID, which links the OMERO dataset to the metadata recorded in eLabFTW.

One of the key features of this workflow step is the automated extraction and implementation of metadata from different disciplinary contexts via their respective eLabFTW entries, followed by annotation at the appropriate organizational level in the OMERO. As a result, metadata do not need to be manually exchanged between systems or annotated redundantly by multiple users. Instead, researchers working with image data gain access to consolidated metadata from biological experiments, plasma treatments, and microscopy acquisitions at a single location aligned with OMERO’s organizational hierarchy. This process is illustrated in Fig. 5, which demonstrates how the Jupyter Notebook interface enables users to annotate image data in OMERO by specifying screen names, plate names, and metadata identifiers retrieved from eLabFTW. As shown, users enter metadata into predefined fields provided by the Jupyter Notebook (Fig. 5) corresponding to OMERO’s organizational levels (“Screen”, “Plate”, and “Well”). Once populated, the notebook automatically establishes links between the eLabFTW metadata (e.g., experiment identifier, operator, and date) and the corresponding image containers in the OMERO server for the annotation of metadata.

After image import and annotation, OMERO maps image acquisition metadata to the OME data model^36^, translating file-specific parameters into a standardized structure. These parameters are stored as key-value pairs (KVPs)^9^, which are required for the annotation of each image and its corresponding experimental context. To incorporate domain-specific metadata from eLabFTW, Adamant, and the Micro-Meta App (see Metadata section), the workflow uses OMERO’s flexible annotation mechanisms, including tag-based and KVP annotations (Fig. 4). KVP annotations encode metadata as explicit attribute-value pairs, allowing biological, plasma, and imaging-relevant parameters to be captured in a semantically consistent manner. For example, datasets are annotated with entries such as cell organism = A375, treatment regime = argon (Ar), cell treatment time [s] = 20, cell treatment distance [mm] = 8, and channel 1 label = DAPI. In parallel, tag annotations are applied at the “Screen”, “Plate”, and “Well” levels to label datasets with key experimental attributes such as cell line, plasma treatment, or time point, enabling intuitive navigation and filtering across imaging studies.

### Data processing

The workflow described in this study focuses on the management, annotation, and organization of bioimaging data and their metadata. Once the datasets are annotated within OMERO, they can be accessed via the OMERO server using downstream image analysis workflows^42^ by means of APIs and associated software plugins, such as FIJI/ImageJ^43^. Using an authenticated connection to the OMERO server, users can retrieve image datasets and their associated metadata for further analysis. FIJI/ImageJ supports batch processing of images and the return of processed images, regions of interest (ROIs), and quantitative results, such as cell counts and intensity measurements, to OMERO^44^. Quantitative results can be stored as KVPs in OMERO or linked back to experimental records in eLabFTW for consistency between raw data, metadata, and analysis outputs. FIJI-OMERO serves as an example of how downstream analysis workflows can be coupled with the data management workflow presented here. Similar approaches can be applied to other analysis environments, such as ImageJ integrated into KNIME^45^ and Galaxy^21^, which support communication with OMERO through their respective software interfaces. Generally, Python-based scripts can be used for custom image analysis and feature extraction, whereas FIJI is used for macro-based batch processing and interactive image analysis^46^.

## Data Sharing

The primary purpose of the presented workflow extends beyond local data management by supporting the generation of bioimaging datasets suitable for sharing and publication. By associating imaging data with experimental metadata through KVP annotations in OMERO datasets, this workflow establishes persistent links between microscopy data, experimental procedures, sample information, gas plasma treatment parameters, and analysis outputs throughout the data life cycle. The workflow establishes (meta)data sharing at multiple levels. Within eLabFTW, experimental records, protocols, and associated metadata can be shared among defined user groups and institutional environments. OMERO offers controlled access to image datasets and annotations, providing collaborative work within research groups and institutions, and public access where configured^19^. These software components facilitate the organization and contextualization of imaging data and associated metadata. The use of JSON-based metadata via Adamant and community-aligned schemas via Plasma-MDS further supports interoperability and the generation of dataset descriptions. Such metadata can be exported and combined with imaging data to facilitate deposition in data repositories for dataset publication and long-term accessibility of the data. In addition, the workflow implements the preservation of raw and processed image data, together with their associated metadata, for future access and interpretation of experimental datasets.

To demonstrate the practical implementation of the presented workflow, we applied it to a representative plasma medicine bioimaging study. The resulting example dataset combines microscopy images, experimental metadata, plasma treatment information, and microscopy acquisition parameters within a single dataset. The example covers the complete workflow, from image and metadata acquisition to the publication of the final dataset in the INPTDAT repository^35^. The following section describes the implementation of the workflow and the generation and publication of example datasets.

## Example Use Case

To illustrate the practical application of the proposed workflow from data acquisition to publication and support its adoption by the community, we applied the workflow to a representative plasma medicine bioimaging experiment. In this experiment, A375 melanoma cells were cultured *in vitro*, treated with gas plasma using kINPen MED^47^ under defined exposure conditions, and subsequently imaged using an automated ZEISS Axio Observer Z1 microscope. The resulting dataset comprised microscopy images, biological metadata, plasma treatment metadata, and microscopy acquisition information. The workflow is executed using the Jupyter Notebook published in WorkflowHub^37^.

**Fig. 6:**
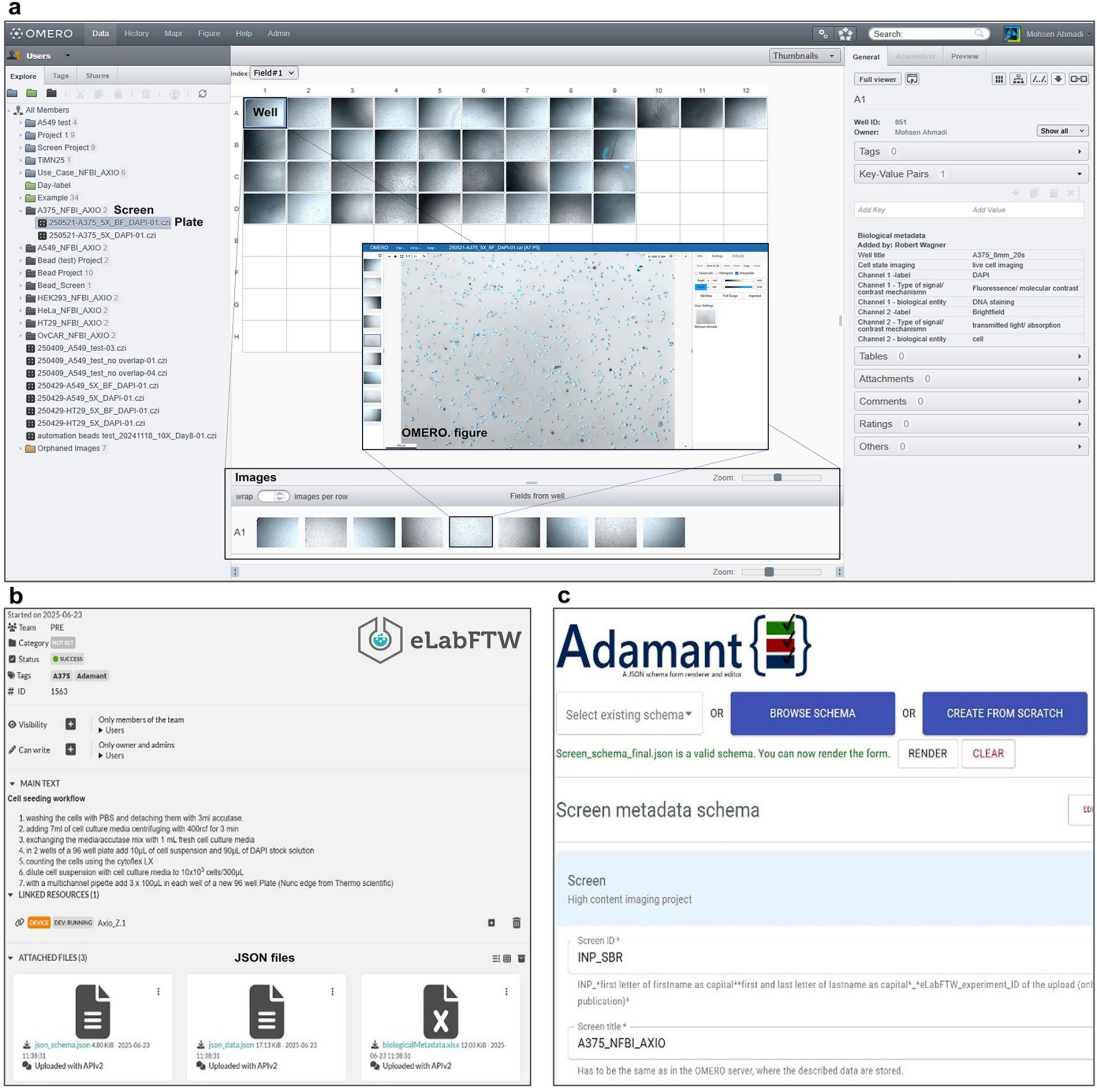
Example workflow in the OMERO user interface illustrating the dataset hierarchy, metadata panel, and preview window. (**a**) The OMERO interface displays an automation-assisted fluorescence imaging plate, including plate layout, well-level image previews, and a representative image field. Biological metadata associated with each well are shown in the metadata panel on the right, (**b**) An eLabFTW entry documenting the corresponding experimental procedure, linked resources, and attached JSON and CSV files that reference the imaging workflow, (**c**) The Adamant interface is used to define and validate the screen metadata schema. Full access to the dataset, including guiding documentation and all materials required to reproduce the example use case, is provided by INPTDAT^35^.

The workflow begins by establishing an authenticated connection to the OMERO server and eLabFTW using their respective APIs. Within the Jupyter Notebook, users provide their OMERO credentials and an eLabFTW API token, which are used to securely access image datasets and experimental metadata. This step is necessary to ensure that all subsequent operations (metadata retrieval, annotation, and validation) of the workflow are carried out within the given access permissions. Next, the user specifies the target OMERO objects for annotation. In this example, a “Screen” representing an imaging experiment is selected. The screen contains multiple “Plates” corresponding to 96-well imaging experiments, each comprising “Wells” that represent individual plasma-treated and untreated conditions. Metadata are captured and annotated at the “Screen”, “Plate”, and “Well” levels within OMERO using persistent identifiers (IDs) that reflect the experimental structure of plasma medicine bioimaging studies. These identifiers enable persistent linkages between the data and metadata (Table 1). These identifiers of the specific “Wells”, “Plates”, and “Screens” are entered once into predefined notebook fields and reused throughout the workflow (Fig. 6a). Using authenticated APIs, the notebook retrieves the metadata stored in eLabFTW as JSON records (Fig. 6b). These metadata are generated during the experiment using Adamant and include information on biological samples, plasma treatment parameters (e.g., plasma treatment time and distance), and experimental design (Fig. 6c). The recorded JSON files in eLabFTW are mapped to the OMERO hierarchy, where “Screen” metadata describe the overall study, “Plate” metadata define the experimental layout, and “Well” metadata contain treatment-specific information (Fig. 6a). In the annotation step, the notebook attaches the selected metadata as KVPs (as represented in Table 1) to the OMERO hierarchy via the provided scripts. These annotations link image data to their biological, plasma, and imaging contexts in a machine-readable form.

**Table 1:** Hierarchical “Screen”, “Plate”, and “Well” metadata for the example use case.

Key	Value
**Screen metadata (collected with Admant)**	
screenID	INP_SBR
screenTitle	A375_NFBI_AXIO
screenAuthor	Author(s)
screenDescription	Treated cells with plasma under different height and time conditions
screenDate	2025-05-21
**Plate metadata (collected with Admant)**	
plateID	INP_SBR
plateTitle	250521-A375_5X_DAPI
description	Treated cells with plasma under different height and time conditions
cellOrganism	A375
mediaType	RPMI (Roswell Park Memorial Institute medium)
mediaVolume [μL]	300
Additive 1	FCS (Fetal Calf Serum)
volumeOfAdditives 1 [μL]	50
Additive 2	L/Glutamine
volumeOfAdditives 2 [μL]	50
Additive 3	Penicillin
volumeOfAdditives 3 [μL]	50
Additive 4	Streptomycin
volumeOfAdditives 4 [μL]	50
seedingDensity [cells/mm^2^]	33.3
layerDescription	Monolayer
plateDate	2025-05-21
creatorName	Author(s)
plateFeatures	Thermo Scientific NUNC Edge 96 well plate
imagingWithLid	True
microscopeName	Axio Observer Z1
**Well metadata (collected with REMBI-compatible Excel template)**	
Well title	A375_8mm_20s
Cell state imaging	Live cell imaging
Channel 1 - Label	DAPI
Channel 1 - Type of signal/contrast mechanisms	Fluorescence/ molecular contrast
Channel 1 - biological entity	DNA staining
Channel 2 - Label	Brightfield
Channel 2 - Type of signal/contrast mechanisms	Transmitted light/ absorption
Channel 2 - Biological entity	Cell
Extrinsic variable used? y/n	n
Treatment regime	Argon (Ar)
Plasma source name	kINPen Med
Cell treatment time [s]	0, 20, 60, 180
Cell treatment conditions	Room temperature and moisture
Cell treatment distance [mm]	8, 12, 16, 20

Once completed, microscopy images are associated with biological metadata, plasma treatment parameters, microscopy acquisition metadata, and provenance information within OMERO. The annotated dataset remains searchable, reusable, and accessible for downstream analysis. The dataset was subsequently deposited in the INPTDAT research data repository^35^. This published dataset serves as a reference implementation of the workflow and demonstrates the complete process from data acquisition and metadata annotation to data sharing and reuse.

## FAIR Assessment

To assess the alignment of the presented workflow with the FAIR data principles defined by Wilkinson *et al*.^10^, we evaluated its implementation across the entire bioimaging data lifecycle, from metadata collection and image management to data sharing and publication. A summary of the assessment is provided in Table 2, and the implementation of the individual FAIR principles is described in the corresponding workflow sections below.

**Table 2:** Summary of the Findable, Accessible, Interoperable, and Reusable (FAIR) assessment of the proposed workflow.

Principle	Workflow implementation
F1. (meta)data are assigned a globally unique and persistent identifier	Persistent identifiers link image datasets, experimental records, and metadata across the workflow
F2. data are described with rich metadata (defined by R1 below)	Rich metadata describes plasma treatments, biological experiments, microscopy settings, and image datasets
F3. metadata clearly and explicitly include the identifier of the data it describes	Metadata explicitly references the corresponding image datasets by persistent identifiers
F4. (meta)data are registered or indexed in a searchable resource	Image datasets and metadata are indexed and searchable within OMERO
A1. (meta)data are retrievable by their identifier using a standardized communications protocol	Data and metadata are accessible through the web interfaces and APIs provided by eLabFTW and OMERO
A1.1 the protocol is open, free, and universally implementable	HTTPS is used as a secure and universal communication protocol throughout the workflow
A1.2 the protocol allows for an authentication and authorization procedure, where necessary	User authentication and permission-based access control provided by eLabFTW and OMERO protect restricted datasets
A2: metadata are accessible, even when the data are no longer available	Metadata remains accessible independently of associated image data
I1. (meta)data use a formal, accessible, shared, and broadly applicable language for knowledge representation	Metadata are exchanged in JSON format, and image data are managed using community-supported formats such as TIFF and OME-TIFF
I2. (meta)data use vocabularies that follow FAIR principles	Community standards and controlled vocabularies support semantic interoperability
I3. (meta)data include qualified references to other (meta)data	Qualified links connect image data with plasma-treatment, biological, and microscopy metadata
R1. meta(data) are richly described with a plurality of accurate and relevant attributes	Datasets are enriched with comprehensive experimental, technical, and imaging metadata
R1.1 (meta)data are released with a clear and accessible data usage license	Datasets can be associated with usage licenses during publication or repository deposition
R1.2 (meta)data are associated with detailed provenance	Provenance is documented through linked experimental records, timestamps, user logs, and analysis outputs
R1.3 (meta)data meet domain-relevant community standards	The workflow aligns with community standards, including OME, REMBI, and Plasma-MDS

The table provides a concise overview of how each FAIR principle according to^10^ (left column) is addressed by the integrated data management workflow for bioimaging in plasma medicine (right column).

### Metadata

The metadata level of the workflow addresses the FAIR principles by systematically using discipline-appropriate ontology-enriched metadata schemas across plasma treatments, biological assays, and microscopy imaging. Metadata are validated according to JSON schemas and exported in JSON format, supporting standardized knowledge representation (I1). Community standards and controlled vocabularies, including REMBI for bioimaging metadata^32^ and ChEBI for chemical entities^48^, improve semantic interoperability (I2) and contribute to rich metadata descriptions (R1). Metadata generated in eLabFTW, Adamant, and the Micro-Meta App are consolidated in OMERO (Fig. 2), where experimental records and image datasets are assigned persistent identifiers (F1). Metadata explicitly reference the associated image datasets (F3), whereas the integration of plasma treatment, biological, and microscopy metadata establishes qualified links between related metadata entities (I3) and provides rich contextual descriptions (F2 and R1). Metadata are exchanged using JSON files and APIs, supporting controlled access (A1), while their storage within OMERO supports continued metadata availability independent of the associated image data (A2).

### Data handling

The data handling level in OMERO provides a technical foundation for implementing FAIR principles across the workflow. OMERO assigns persistent identifiers to datasets^9^ (e.g., “Screen” and “Plate” IDs; F1), explicitly links metadata to the corresponding image datasets (F3), and supports rich metadata annotation (F2). Datasets and associated metadata are indexed and searchable via the OMERO interface (F4). Metadata and image data are accessible via the OMERO web interface and open APIs (A1), which are based on standardized and widely implementable communication protocols (A1.1). User authentication and project-based access control support secure access where required (A1.2). Metadata remains available independently of the associated image data, supporting continued metadata accessibility (A2). Interoperability is achieved by utilizing machine-readable (meta)data formats (e.g., JSON, TIFF, and OME-TIFF; I1) and qualified links between related metadata entities (I3). Finally, the workflow establishes data reuse by collecting experimental and technical metadata (R1), optional dataset licensing (R1.1), provenance information recorded by user logs and timestamps (R1.2), and alignment with community standards, including OME, REMBI, and Plasma-MDS (R1.3).

### Data processing

At the data processing level, the FIJI-OMERO example illustrates how annotated datasets generated by the workflow can be accessed using authenticated OMERO connections (A1 and A1.2). Images are retrieved via the OMERO API, processed using reusable FIJI macros, and analysis outputs, including ROIs, quantitative measurements, and processed images, can be returned to OMERO, together with annotations. This preserves the links between image data, metadata, and analysis results (F3 and I3), records analysis provenance (R1.2), and stores results in machine-readable formats (I1). Reusable macros support the batch processing of OMERO-hosted datasets while maintaining the association between raw images, metadata, and analysis outputs. This demonstrates how downstream image analysis can be integrated into the proposed workflow while preserving the documentation and traceability.

### Overall FAIR assessment of the workflow

Taken as a whole, the workflow demonstrates a coherent and end-to-end implementation of the FAIR principles across metadata capture, data handling, and data processing. Findability is set by applying persistent internal identifiers, organizational hierarchies, and comprehensive indexing, which allow metadata and image data to be reliably located and referenced throughout their life cycle. Accessibility is supported by network-based access mechanisms combined with authentication and authorization controls, while preserving metadata availability beyond the lifetime of the raw image data. Interoperability is achieved at the technical level by consistently using machine-readable formats, standardized schemas, and explicit semantic links between related entities, such as image dataset ↔ biological sample, plasma treatment, and microscopy acquisition settings; OMERO dataset ↔ eLabFTW experiment; and experimental record ↔ metadata JSON file. Reusability is enabled by the rich contextualization of data using community-aligned metadata standards and maintaining traceable links between experiments, images, and derived results, such as quantitative measurements, ROIs, processed images, and analysis outputs. In the FAIR assessment of the workflow, the Jupyter Notebook located on GitHub contains all the required components for data curation and annotation and serves as a practical implementation of the workflow. This makes the workflow steps reproducible, links data and metadata across OMERO, eLabFTW, and Adamant by means of APIs, implements standardized schemas in machine-readable form, and records provenance, parameters, and versions. The workflow does not constitute a repository or published FAIR object but operationalizes FAIR principles through metadata standardization, integration, and annotation and demonstrates these concepts using a publicly available example dataset.

**Fig. 7:**
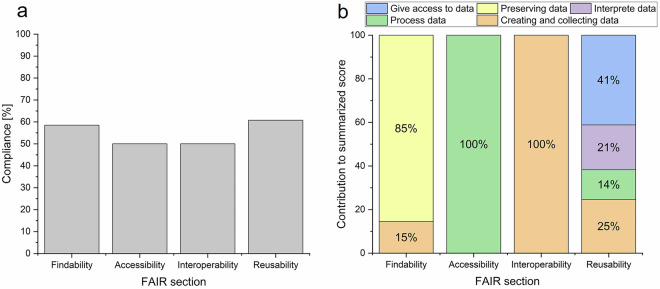
Summary report generated with the Data Stewardship Wizard using the Common DSW Knowledge Model 2.6.10. (**a**) average FAIR compliance scores of the implemented data management workflow for bioimaging in plasma medicine; (**b**) contribution of selected questionnaire domains to the summarized FAIR scores. The corresponding knowledge model is available at: https://registry.ds-wizard.org/knowledge-models/dsw:root:2.6.12.

Although certain aspects, such as vocabulary refinement and extended provenance tracking, remain subject to iterative improvement, the workflow demonstrates the practical implementation of FAIR principles and provides an extensible framework for bioimaging data management in plasma medicine. The FAIR principles define high-level objectives for data management but do not provide detailed, domain-specific guidance on how these objectives should be implemented in practice. Therefore, the workflow was first evaluated according to the FAIR principles, and the results of this assessment were subsequently used to complete the Common DSW Knowledge Model questionnaire (see the Method section). The DSW FAIR assessment is used to determine which levels of the workflow contribute to overall FAIR compliance^38^. This offers an organized evaluation of how metadata, data handling, and data processing levels contribute to the partial/operational FAIR alignment of the workflow. Figure 7 summarizes the resulting FAIR assessment and the contribution of individual questionnaire topics to each dimension. Figure 7a shows the compliance scores for each FAIR section, indicating moderate (50–60%) fulfilment of findability, accessibility, interoperability, and reusability. The limitations in the area of accessibility are primarily because access to stored microscopy images, i.e., particularly those involving patient samples from partner hospitals, must be individually regulated. As a result, full accessibility compliance cannot be fully achieved until user-specific access requirements are more clearly defined. Figure 7b shows how different activity domains within the workflow contribute to these scores. The findability score is primarily driven by data creation and collection, whereas accessibility and interoperability are fully supported by processes related to granting access to data and preserving data. In contrast, reusability is influenced by a broader combination of activities, including data interpretation, creation and collection, processing, and preservation, reflecting its dependence on complete and semantically rich metadata. This variation originates from the questionnaire’s adaptive structure; depending on prior responses, different follow-up questions may be triggered or omitted, leading to workflow-specific differences in assessment depth and emphasis.

## Conclusion and Outlook

We introduced a data management workflow for bioimaging in plasma medicine that supports the implementation of the FAIR principles for the handling and sharing of image datasets. The workflow integrates image acquisition, experimental documentation (including gas plasma treatment and biological assays), and metadata annotation across OMERO, eLabFTW, Adamant, and the Micro-Meta App, by means of API-based connections within a Jupyter Notebook environment. The workflow notebook and accompanying documentation published in WorkflowHub are required to reproduce the workflow described herein. It includes example scripts for OMERO-eLabFTW API integration, retrieval of experiment and dataset identifiers, OMERO authentication, eLabFTW connectivity, and image dataset annotation.

Using Jupyter Notebook as a widely adopted research environment, the workflow introduces guided access to existing and open-source data management tools, metadata standards, and annotation procedures without requiring extensive programming expertise. Using a plasma medicine bioimaging experiment, we demonstrated how the workflow establishes data management by associating the imaging data with experimental and technical metadata. The resulting dataset was published in the INPTDAT repository for sharing and reusability.

In addition, a structured FAIR assessment was used to evaluate the workflow across the FAIR dimensions of findability, accessibility, interoperability, and reusability. The resulting analysis identified the extent to which individual workflow stages, including metadata collection, data handling, and data processing, complied with FAIR principles up to a certain level, and pointed out areas in which further development and broader community adoption are required.

The published dataset offers a reference implementation of the workflow and serves as a basis for future benchmarking and reuse. The workflow has not yet been systematically evaluated across multiple laboratories or repository environments. Nevertheless, published example datasets provide a foundation for community adoption and future studies by establishing standardized metadata collection, annotation procedures, and the integration of plasma treatment and imaging data.

Ongoing developments within initiatives such as NFDI4BIOIMAGE and Plasma-MDS are expected to expand the metadata standards adopted by the workflow, including controlled vocabularies, ontology mapping, and plasma-specific metadata fields. Technical interoperability can be achieved by utilizing standardized image formats such as OME-TIFF and OME-NGFF^49^ and JSON-based metadata exchange, whereas semantic interoperability is supported by controlled vocabularies and community data models, including the OME data^36^ and Plasma-MDS^34^ models. These initiatives further support interoperability, publication, and the provision and use of plasma medicine bioimaging datasets.

## Data Availability

The example plasma medicine bioimaging dataset, including supporting documentation, metadata records, and materials required to reproduce the use case presented in this study, has been deposited in the INPTDAT research data repository and is publicly accessible^35^. The included image database contains all raw images in.czi and TIFF/XML format.

## Code availability

The Jupyter notebook-based implementation of the workflow is publicly accessible via the project’s GitHub repository (https://github.com/INP-SDT/Bioimaging-Data-Management-Workflow-for-Plasma-Medicine) and published in WorkflowHub^37^.
